# Synthesized diterpene lactone derivative attenuated Freund’s complete adjuvant-induced arthritis in Wistar rats

**DOI:** 10.22038/IJBMS.2024.75023.16295

**Published:** 2024

**Authors:** Patrick Francis Kimariyo, Sony Priya Kurati, Perupogu Suvarna Babu, Alfredi Alfred Moyo, Murali Krishna Kumar Muthyala

**Affiliations:** 1 AU College of Pharmaceutical Sciences, Pharmaceutical Chemistry Department, Andhra University, Visakhapatnam, Andhra Pradesh 530003, India; 2 Dar es Salaam Institute of Technology (DIT), Science and Laboratory Technology Department, Bibititi and Morogoro Rd Junction P. O. Box 2958. Dar es Salaam, Tanzania; 3 Medicinal Chemistry Research Laboratory, Department of Chemistry, Shivaji University, Kolhapur 416004, Maharashtra, India; 4 Mabibo Traditional Research Centre, National Institute for Medical Research (NIMR), Barack Obama Drive, P.O.Box 9653, 11101 Dar es Salaam, Tanzania

**Keywords:** Anti-arthritic, Anti-rheumatoid, Clerodane diterpene, FCA model, Rheumatoid arthritis

## Abstract

**Objective(s):**

In this study, the SP-38 (Diterpene Lactone derivative) was designed, synthesized from clerodane diterpene (lactone) isolated from *Polyanthia longifolia* var. pendula, and tested for anti-arthritic activity using the FCA-induced arthritic rat model.

**Materials and Methods:**

This study examined the *in vivo* effects of SP-38 using three different doses (20, 10, and 5 mg/kg) by oral administration for 21 days from day 8 after 0.1 ml FCA sub-planter injection until day 28. Arthritis index, paw swelling, ankle diameter, body weight as well as biochemical, hematological, histopathological, and radiological parameters were examined.

**Results:**

Administered SP-38 reduced arthritis index, paw volume, and joint swelling compared to the arthritic control group. Accordingly, rats treated with SP-38 showed a remarkable increase in body weight and improved biochemical, hematological, histopathological, and radiological parameters. Furthermore, it reduced the increased production of CRP and RF while simultaneously decreasing ESR in all SP-38-treated rats. However, SP-38 showed promising liver protection by reducing elevated serum levels of liver and kidney function markers SGOT, SGPT, and ALP. Furthermore, splenic index, TNF-α, and IL-6 levels were significantly reduced compared to arthritic control rats at certain doses.

**Conclusion:**

The result of the present study concludes that SP-38 has significant anti-arthritic potential in FCA-induced arthritis in Wistar rats. SP-38 therefore showed promising anti-arthritic activity, as evidenced by attenuation of inflammation, inflammatory markers, and pro-inflammatory cytokine levels.

## Introduction

Rheumatoid arthritis (RA) is a long-lasting autoimmune inflammatory disease that results in the destruction of limb joints and progressive damage to secondary organs (1). It explains that RA is a chronic systemic autoimmune inflammatory disease characterized by persistent joint inflammation leading to cartilage and bone damage, disability, and ultimately systemic disease (2, 3). 

Currently, Methotrexate (MTX) is documented as the gold standard for the treatment of RA, including its enhancements, even at the nano level (4, 5). The MXT drug is being proposed as the foremost disease-modifying anti-rheumatic drug (DMARD) and despite its recommendation along with other biological DMARDs, it still shows some resistance and some toxicity for some patients (6, 7). For this reason, the anti-rheumatoid arthritis activity has been ascribed to natural compounds, but other synthesized compounds such as purpurin and coumarin have recently been shown in pre-clinical studies to have the same effects (8, 9).

**Figure 1:**
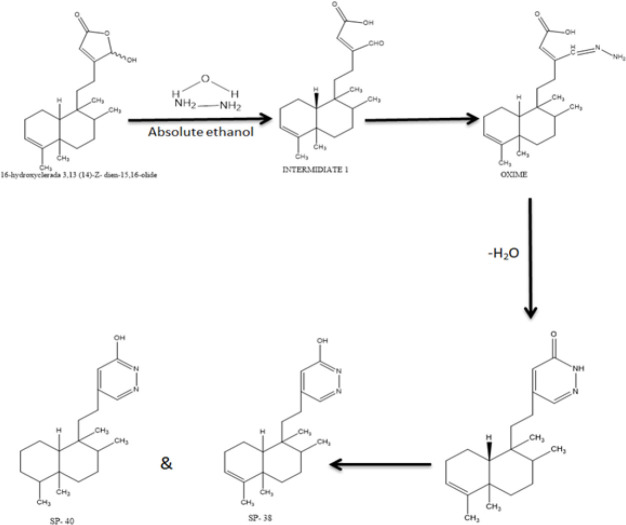
Scheme for structural derivation of SP-38

A clerodane diterpene (Lactone) which was explained to have ant-inflammatory activity attracted our attention for structural modification to be screened for anti-rheumatoid activity (10). The team hypothesized that molecular modification of this natural lead compound is important in designing new anti-arthritic drugs as it was observed when tested for anti-mycobacterial activity (11). Structural modification and derivatization of compounds are warranted to improve the absorption, distribution, metabolism, and excretion and or activity of active compounds as documented by previous studies that utilized semi-synthesized compounds such as glycosylated flavonoids, enones and purpurin that resulted into activity enhancement (8, 12, 13). The practicality of enhancing biological activity through structural modification and/or derivatization of compounds for RA is justified by a few examples such as that of enones and coumarin (14, 15). Therefore, the primary objective of our study is to investigate the anti-rheumatoid arthritis of our semi-synthesized compounds using FCA induced rat model. In order to increase the number of compounds for anti-rheumatoid arthritis activity screening, we took (clerodane diterpene) lactone as the lead compound and modified its structure as shown in the scheme for structural derivatization (Figure 1). Two nitrogen atoms from hydrazine hydrate were substituted in the molecule while maintaining its skeleton structure. Then, the semi-synthesized compound underwent *in vivo* screening for anti-rheumatoid arthritis.

## Materials and Methods

***Reagents and chemicals***

FCA (Freund’s complete adjuvant- heat-killed *Mycobacterium* tuberculosis suspended in paraffin oil and mannide monooleate 10 mg/ml, Cat. No 7027) was purchased from Chondrex Inc. (Redmond, WA, USA), TNF-α, #KB3145 and IL-6, #KB3068 (Krishgen Biosystems, Mumbai, India), Methotrexate, Di-ethyl ether, and Paraformaldehyde were from Sigma-Aldrich Chemical Company were purchased from India Cash and Carry Pvt. Ltd. RF, SGOT, SGPT and ALP kits from DELTALAB India and all other solvents and reagents utilized in the study were of analytical grade and procured from authentic vendors.

***Design and synthesis of compound (SP-38)***

The synthesis of semi-synthetic heterocycles SP-38 from the clerodane diterpene called lactone (16α -hydroxycleroda-3, 13 (14) Z-dien-15, 16-olide) isolated from the plant *Polyalthia longifolia *var *Pendula.*

A mixture of 1.0 eq (0.3 mM) 16-hydroxycleroda3,13 (14)-dien15,16-olide (Lactone; clerodane diterpenoid) and 2.5 equivalent (0.75 mM) 16α-hydroxycleroda-3,13 (14) Z-dien-15,16- olide & hydrazine hydrate was well-maintained in 5 ml of absolute ethanol at room temperature. To monitor the reaction’s progression TLC was used, ethanol alcohol was evaporated after the reaction, and the product was extracted using ethyl acetate and water. The ethyl acetate layer was concentrated by using column chromatography with silica gel and purified by sequentially eluting 2.5 liters of n-hexane, ethyl acetate, and methanol. At 5% ethanol: hexane proportion, a pale-yellow crystalline powder was formed. Crystals were refined by recrystallizing them in hexane, and then they were dried. The structure of the freshly synthesized compound was determined by using a variety of mass spectrometry techniques, including IR, 1D, 2DNMR, and MASS spectral data. The spots were visualized using iodine, UV, and acid spray, respectively. EZMELT 120 (Stanford Research Systems, USA) was used to determine the uncorrected melting points. The Bruker ALPHA-T FTIR device was used to gather IR spectra data using the KBr pellet technique. Using TMS as an internal standard, NMR spectra data were collected on a Bruker-400 MHz machine using the proper deuterated solvent. Studies on elemental analysis were carried out utilizing a Carlo Erba elemental analyzer. Agilent 6410 QQQ MS gear was used to perform ESIMS mass spectral observations.

***Toxicity study of semi-synthesized compounds***

The acute toxicity study was performed according to the OECD guidelines 423 (16). Adult Wister rats of both sexes of 150 to 200 grams grouped in 4 groups each with 3 animals were used in the study. All rats were fasted overnight and provided water *ad libitum*. After the fasting period, the test compound was orally administered at a dose of 2000 mg/kg body weight following the requirements of OECD guidelines number 423 (16). Rats were observed individually after being dosed at regular intervals for the first 30 min to the first 24 hr, with particular attention being paid for the first 4 hr and daily thereafter for a total of 14 days. The focus was on observing convulsions, tremors, diarrhea, lethargy, salivation, sleep, and coma. After no death was observed, the study was repeated at the same dose to approve the results. Finally, based on the mortality results, the rats were treated with much lower doses of SP-38 (20 mg/kg, 10 mg/kg, and 5 mg/kg). These 20, 10, and 5 mg/kg doses were selected based on the previous similar studies that involved the administration of different anti-rheumatoid compounds (13, 17-21).

***Experimental animals and housing***

Andhra University (AU) College of Pharmacy Science (Visakhapatnam): Seven-week-old healthy Wister rats (n=36) 150-180 g were considered for the experiment from Mahaveera Enterprises, Telengana, India. Upon arrival, the animals were housed in open cages at 25±2 ^°^C with a 12:12 hr dark/light cycle. Animals’ acclimatization to the laboratory settings was done for 7 days before the start of the experiments, and they were given *ad libitum* access to standard pellet chow and water. 

***Experimental design***

This assay was carried out according to that previously described by Zia *et al*. (2022) with some modifications(22).

The experimental design utilized a total number of 36 animals in six groups (n=6) as follows:

Group I: Normal Control (Equal volume of distilled water),

Group II: Arthritic Control (Equal volume of distilled water),

Group III: Standard Control (Methotrexate 0.5 mg/kgbw), Group IV: SP-38 (20 mg/kgbw),

Group V: SP-38 (10 mg/kgbw),

Group VI: SP-38 (5 mg/kgbw).

On day 0, all groups were injected with 0.1 ml FCA in the sub-plantar area in the right hind paw, except for the normal control group, which received 0.1 ml paraffin oil.

The test compounds and the standard drug (MTX) were suspended in CMC. The animals were treated daily beginning with day 8 (phase of arthritis development) to the 28^th^ day continuously as previously described by Amin *et al*. (2022)(23). On the following days 0, 4, 8, 12, 16, 20, 24, and 28, several parameters including paw volume, arthritis score, ankle joint diameter, and body weight were recorded. At the end of dosing administration on day 28, the animals were sedated with diethyl ether, and blood collection was done by a retro-orbital puncture for the characterization of serum hematological and biochemical parameters as well as pro-inflammatory cytokines (23). The blood collected for biochemical parameters and pro-inflammatory cytokines was centrifuged at 1200 g for 5 min at room temperature and the collected serum was collected (24). The animals were carefully dissected to determine the weight of the spleen, and the FCA-injected ankles were removed for histopathological and radiological analysis.

***Clinical assessment of arthritis and arthritis index***

The arthritic score was set in the range of 4 to 0, whereby 4 is severe deformity of the paw with swelling and erythema and 0 is a normal paw with no swelling or erythema. A maximum score of 8 was set for a total score of two hind paws from each rat as previously described (25).

***Rat ankle diameter (thickness)***

Rat ankles’ thicknesses were measured by using a digital Vernier caliper to determine the effect of different treatments on the ankle diameter size (26). The changes in inflammation (%) were calculated from day 8 using the following formula to compare the differences between the groups: Ankle swelling (%)=((Dt−Dn)/Dn×100%), where Dn and Dt are the diameters of the right ankle before and after FCA injection, respectively in different days as described by Meng *et al.* (2021)(17).

***Rat paw volume/edema***

Paw volumes were measured every 4 days (Et) from day 0 (Eo) utilizing a digital Plethysmometer model No CS-354. The percentage changes of inflammation (%)=((Vt-V_0_)/V_0_×100%), where V_0_ and Vt are the volumes of the right hind paw initially and after inflammation, respectively as described by Meng *et al.* (17).

***Body weight***

The body weights were measured every four days to demonstrate the changes exerted by treatment agents (25).

***Spleen index***

Fresh organ (Spleen) weight was measured directly to compute the immune organ index ((spleen weight in mg/bodyweight in grams) x100) as previously done by Cellat *et al.* (27).

***Animal serum biochemistry (Liver enzymes and inflammatory biomarkers)***

RF and CRP as inflammatory markers were determined using the collected serum. SGOT, SGPT, and ALP since these are good parameters to study if any damage or injury has occurred to the liver as liver toxicity is termed to be the potential side effect of arthritis medications (28). The liver marker enzymes were determined using the automatic biochemistry analyzer Erba EM 200 following instructions from kit manufacturers. ESR was done using a modified Westergren tube using EDTA anticoagulated blood. 

***Determining the effect of SP-38 on hematological parameters***

The freshly collected blood was used for the estimation of complete blood count (CBC) by using a hematology analyzer (Sysmex xp 100). The blood cell count i.e., (WBCs), (RBCs), and platelets was done. 

***Effect on pro and inflammatory cytokines***

Blood was collected from each rat and centrifuged at 3000 rpm for 10 min and the serum was kept at -20 ^°^C until analysis. Two core pro-inflammatory cytokines (TNF-α, and IL-6) in serum were measured using ELISA kits (Krishgen) according to the manufacturer’s instructions. The concentrations of TNF-α and IL-6 were determined from the standard curve.

***Radiological investigation***

On day 28 of the study, radiographs of the excised ankle and lower extremities of the rats were evaluated for changes in the joints, joint space, and soft tissue swelling (29).

***Histopathological examination***

The right limbs were cut from humanely killed rats and 10% buffered neutral formalin was used for fixation. Decalcification was done by dipping the legs in 10% EDTA (pH 7.4) solution at 4 ^°^C for 3 weeks, during which the EDTA solutions were renewed every 4 days, embedded in paraffin wax, sliced in solid sections of 4 µm thickness, and stained with Hematoxylin and Eosin (H&E) for general evaluation (30). Blind investigation of stained slides was made by an independent pathologist for bias minimization. The microscopic arthritic alterations on joint tissue changes were evaluated to determine the severity of the disease. The following scoring system was used: 0 indicated no signs of inflammatory cell infiltration around the joint area; 1 indicated minimal infiltration; 2 indicated slight infiltration; 3 indicated moderate infiltration; and 4 indicated significant infiltration (19). Enlargement in the synovial lining cell layer, synovial hyperplasia, synovial vascularity, pannus formation, cartilage erosion, and bone erosion were also evaluated according to the previous description (23, 31). The histological slide images were captured at two different magnifications (low (×10) and high (×40)) using a microscope camera (Leica DM750) and processed.

***Statistical data analysis***

One-way analysis of variance (ANOVA) with Tukey’s *post hoc* test was used for statistical data analysis. *P*-value<0.05 was considered significant. All the obtained values are expressed in terms of mean±standard deviation (SD), n=6. 

**Table 1:** ^1^H and ^13^C spectral data of compound 1 (SP-38)

**Position**	**Compound 1 (SP-38) NMR data** **(400 MHz, CDCl**_3_ **)**	
	^13^ **C**	^1^ **H**
1	18.31	1.43
2	27.2	2.05
3	120.2	5.22
4	139.5	-
5	38.1	-
6	37.5	1.27-1.37
7	26.78	1.41
8	36.6	1.40
9	38.9	-
10	46.5	1.38
11	36.3	1.50-1.52
12	19.84	2.30
13	126.3	-
14	149.5	6.71
15	161.5	11.20
16	139.3	7.65
17	18.31	0.78
18	16.1	0.86
19	19.6	1.64
20	20.6	1.03

**Table 2:** Physicochemical constants for the Compound (SP-38)

**Compound code**	**Structure **	**Yield (%) **	**M.P. (°C)**	**ESI-MS [M]+** ***m*** **/** ***z***	**Mol. Formula**	**Elemental analysis**		
						**Element**	**REQ**	**FOUND**
**Compound 1 (SP-38)**	5-(2-((8aR)-1,2,4a,5-tetramethyl1,2,3,4,4a,7,8,8a-octahydronaphthalen-1-yl) ethyl) pyridazine-3-ol	75%	195-197	314.47	C_20_H_30_N_2_O	C H O	75.86 9.70 14.44	75.84 9.69 14.42

**Table 3:** Effects of oral administration of SP-38 on the Spleen Index in arthritic rats

Treatment group	Final rat mean weight (gm)	Spleen mean weight (gm)	Spleen index
Normal Control	206.33±2.04	5.54±1.04	3.08±0.55
Arthritic Control	188.1±3.27*	15.83±1.55*	7.93±0.80*
Standard (MTX)	203.03±2.88^#^	7.63±1.52^#^	4.03±0.76*^#^
SP-38(20 mg/kg)	198.50±3.62*^#^	9.04±1.62*^#^	4.56±0.70*^#^
SP-38(10 mg/kg)	197.00±4.29*^#^	9.08±1.66*^#^	4.62±1.74*^#^
SP-38(5 mg/kg)	196.33±3.55*^#$^	9.47±2.18*^#^	4.84±1.64*^#^

All the obtained values are expressed in terms of mean±standard deviation (SD), n=6. * Significantly different from the Normal control group at *P<*0.05. # Significantly different from the arthritic control group at *P<*0.05. $ Significantly different from the Standard (MTX) group at *P<*0.05

**Table 4:** Effects of oral administration of SP-38 on inflammatory markers of Rheumatoid arthritis in arthritic rats

	**CRP (mg/dl)**	**RF (IU/ml)**	**ESR (mm)**
Normal Control	4.17±0.75	9.03±1.17	3.45±0.64
Arthritic Control	25.98±4.00*	28.97±5.71*	8.70±1.54*
Standard (MTX)	7.67±1.63*^#^	13.97±2.04^#^	4.98±0.52^#^
SP-38 (20mg/kg)	16.00±2.83*^#$^	18.00±3.10*^#^	5.77±0.99*^#^
SP-38 (10mg/kg)	18.17±2.14*^#$^	21.17±3.87*^#$^	6.25±1.00*^#^
SP-38 (5mg/kg)	19.17±2.48*^#$^	23.17±1.47*^#$^	6.65±1.07*^#^

All the obtained values are expressed in terms of mean±standard deviation (SD), n=6. * Significantly different from the Normal control group at *P<*0.05. # Significantly different from the arthritic control group at *P<*0.05. $ Significantly different from the Standard (MTX) group at *P<*0.05

**Table 5:** Effects of oral administration of SP-38 on liver marker enzymes in arthritic rats

	**SGOT (IU/L)**	**SGPT (IU/L)**	**ALP (IU/L)**
Normal Control	100.15±13.60	34.02±6.07	148.99±18.35
Arthritic Control	289.93±14.98*	105.95±16.33*	303.03±26.22*
Standard (MTX)	154.01±7.41*^#^	47.98±8.40^#^	182.11±27.74^#^
SP-38(20mg/kg)	193.88±20.21*^#$^	56.15±10.87*^#$^	252.00±28.39*^#$^
SP-38(10mg/kg)	206.19±12.76*^#$^	59.42±6.27*^#$^	549.00±33.98*^#$^
SP-38(5mg/kg)	211.79±10.51*^#$^	67.54±9.11*^#$^	249.33±32.25*^#$^

All obtained values are expressed in terms of mean±standard deviation (SD), n=6. * Significantly different from the Normal control group at *P<*0.05. # Significantly different from the arthritic control group at *P<*0.05. $ Significantly different from the Standard (MTX) group at *P<*0.05

**Table 6:** Effects of oral administration of SP-38 on haematological parameters in arthritic rats

	Normal control	Arthritic control	Standard (MTX)	SP-38 (20 mg/kg)	SP-38 (10 mg/kg)	SP-38 (5 mg/kg)
**WBCs **(x10^3^/uL)	7.10±1.16	17.93±3.52*	8.63±1.48^#^	12.07±3.39*^#^	13.82±1.93*^##^	14.45±1.89*^$^
**RBCs **(x10^6^/uL)	7.62±0.51	5.06±0.99*	6.98±0.38^#^	6.93±1.53^#^	6.45±1.06	6.08±1.43
**HGB **(g/dL)	13.02±1.56	9.10±1.06*	12.93±1.46^#^	12.33±1.14^#^	11.20±1.37	10.68±1.43
**PLTs **(x10^3^/uL)	856.83±111.74	1377.03±134.87*	906.01±122.08^#^	968.50±173.79^#^	1193.00±173.53*^$^	1243.33±1166.47*^$^
**HCT **(%)	40.98±3.61	30.48±1.50*	40.12±2.10^#^	41.05±3.97^#^	37.07±2.17^#^	35.42±3.98*

All obtained values are expressed in terms of mean±standard deviation (SD), n=6. * Significantly different from the normal control group at *P<*0.05. # Significantly different from the arthritic control group at *P<*0.05. $ Significantly different from the standard (MTX) group at *P<*0.05

**Figure 2:**
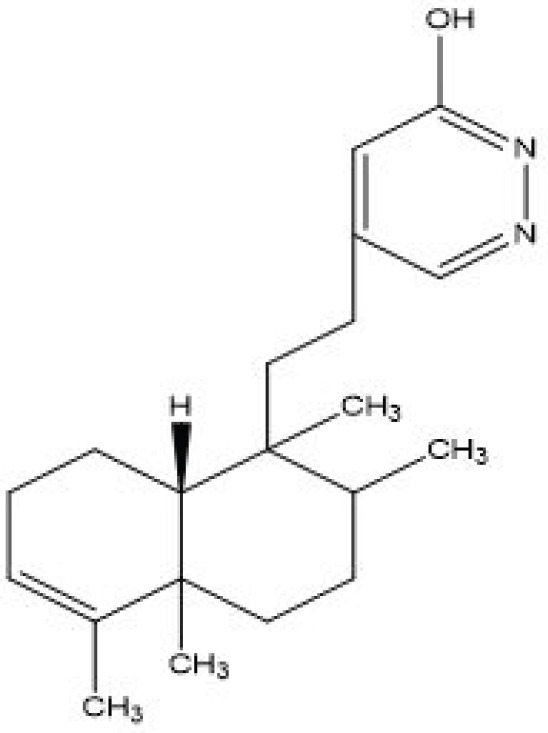
SP-38 compound

**Figure 3:**
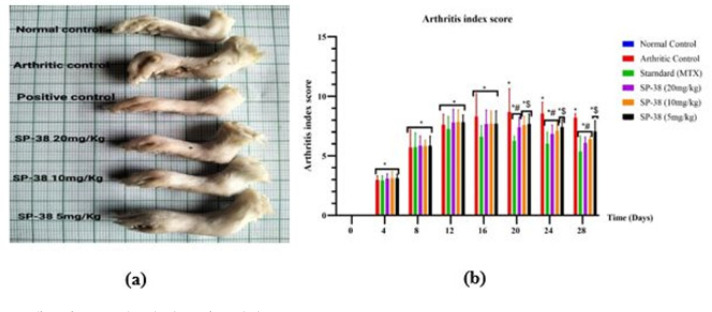
Effects of SP-38 on clinical arthritis after oral administration

(a) Images showing clinical arthritis of right rats’ limbs (b) Arthritis index score in Wister rats from day 0 to day 28. All obtained values are expressed in terms of Mean±Standard Deviation (SD), n=6. *Significantly different from the Normal control group at *P<*0.05. #Significantly different from the arthritic control group at *P<*0.05. $Significantly different from the Standard (MTX) group at *P<*0.05

**Figure 4:**
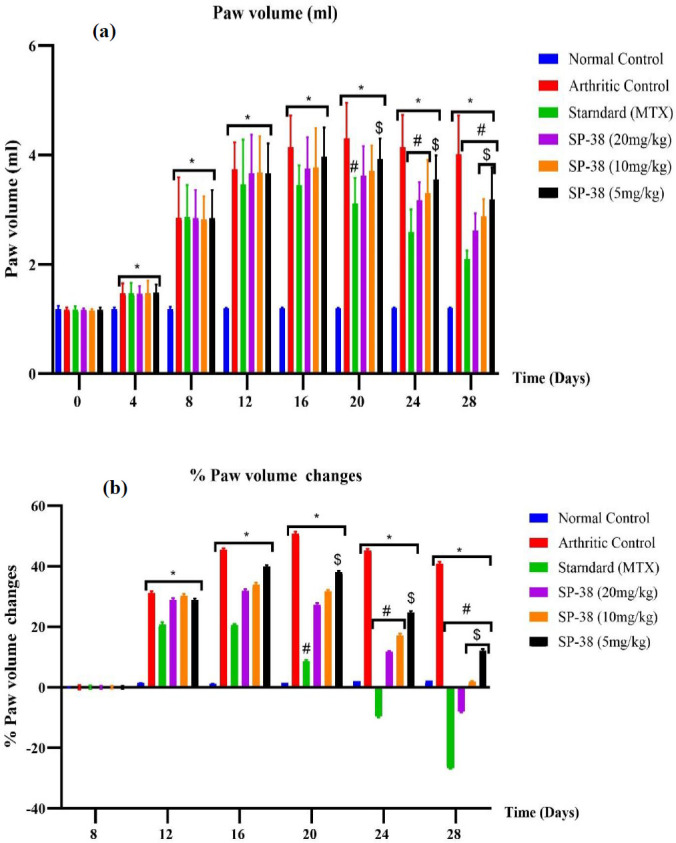
Effects of SP-38 on paw volume changes after oral administration in Wister rats

(a) Paw volume increased from day 0 to day 28. (b) Percentage changes in paw volume from day 8 to day 28. All the obtained values are expressed in terms of Mean±Standard Deviation (SD), n=6. *Significantly different from the Normal control group at *P<*0.05. #Significantly different from the arthritic control group at *P<*0.05. $Significantly different from the Standard (MTX) group at *P<*0.05

**Figure 5:**
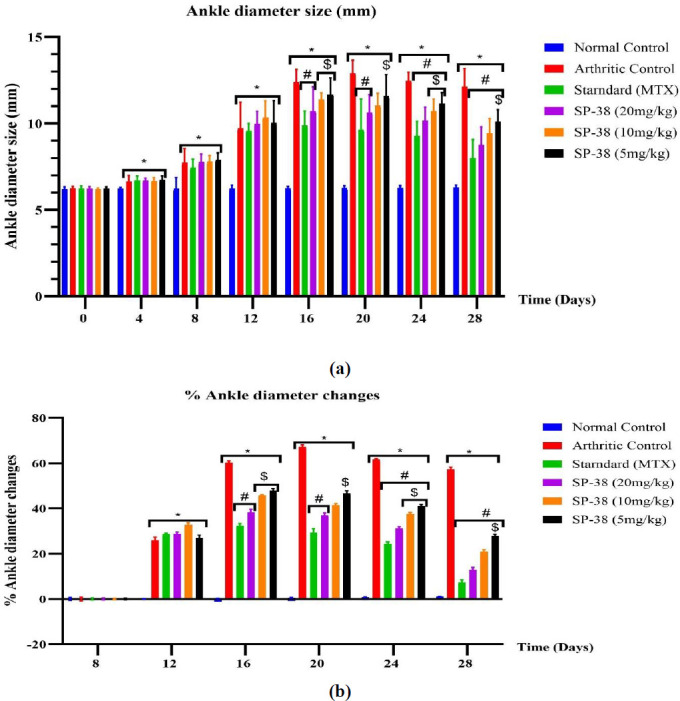
Effects of SP-38 on ankle diameter after oral administration in Wister rats

(a) Ankle diameter increased from day 0 to day 28. (b) Percentage changes in ankle diameter from day 8 to day 28. All obtained values are expressed in terms of mean±standard deviation (SD), n=6. * Significantly different from the Normal control group at *P<*0.05. # Significantly different from the arthritic control group at *P<*0.05. $ Significantly different from the Standard (MTX) group at *P<*0.05

**Figure 6:**
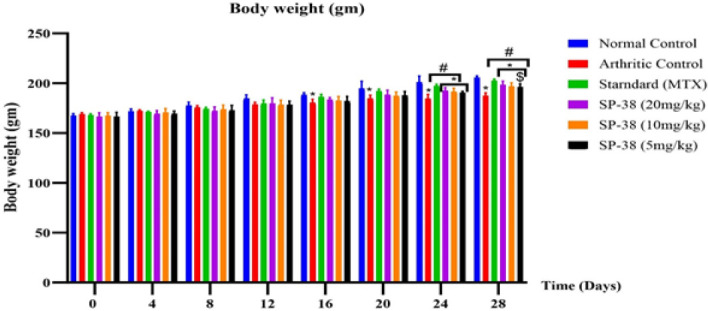
Effects of SP-38 on body weight from day 0 to day 28

All the obtained values are expressed in terms of mean±standard deviation (SD), n=6. * Significantly different from the normal control group at *P<*0.05. # Significantly different from the arthritic control group at *P<*0.05. $ Significantly different from the Standard (MTX) group at *P<*0.05

**Figure 7:**
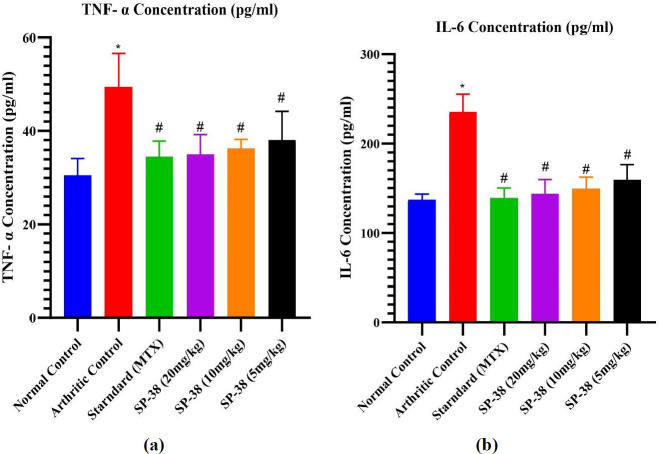
Effects of oral administration of SP-38 on (a) TNF- α and (b) IL-6 in arthritic rats

All the obtained values are expressed in terms of mean±standard deviation (SD), n=6. * Significantly different from the Normal control group at *P<*0.05. # Significantly different from the arthritic control group at *P<*0.05. $ Significantly different from the standard (MTX) group at *P<*0.05

**Figure 8:**
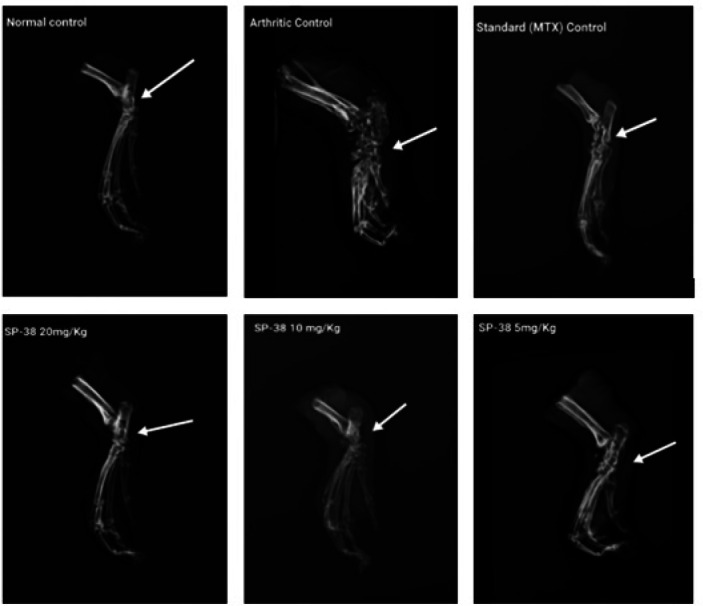
Effects of oral administration of SP-38 on animals’ ankle joint space, and soft tissue swelling in arthritic rats

**Figure 9:**
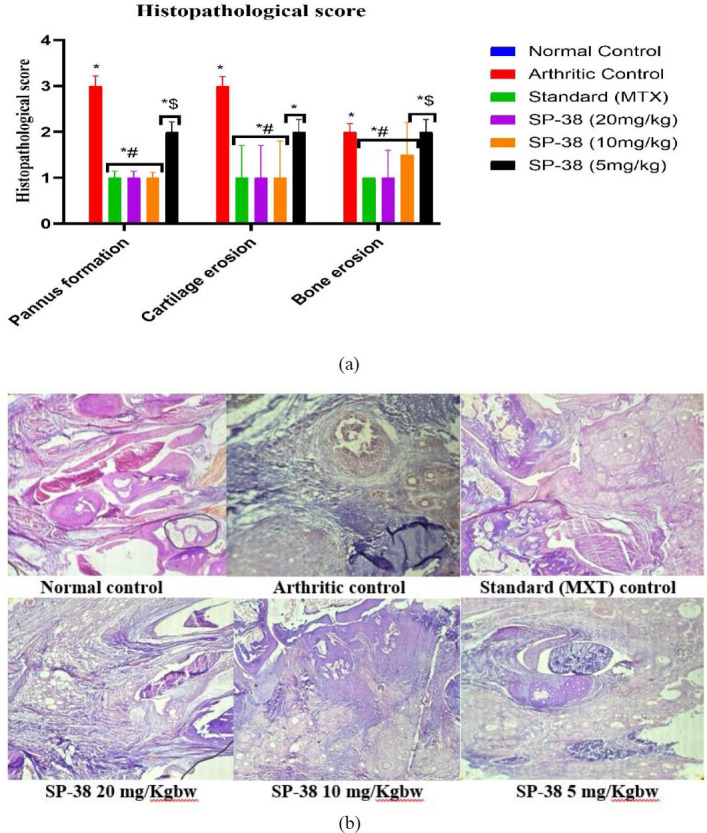
Histopathological ffects of oral administration of SP-38 on animals’ on ankle joint tissues in arthritic rats

(a) Histological scores of the right hind ankle from randomly selected rats in each group scored as previously performed by Yang *et al.* (2018)(31). (b) Histopathology of arthritic rat’s hind paw of immunized Wister rats treated with different doses of SP-38 (40× magnification). All the obtained values are expressed in terms of mean±standard deviation (SD), n=6. * Significantly different from the Normal control group at *P<*0.05. # Significantly different from the arthritic control group at *P<*0.05. $ Significantly different from the Standard (MTX) group at *P<*0.05

## Results

***Chemistry***

Characterization of Compound 1 (SP-38) (5-(2-((8aR)-1,2,4a,5-tetramethyl-1,2,3,4,4a,7,8,8a-octahydronaphthalen-1-yl) ethyl) pyridazin-3-ol:

The Compound (SP-38)(Figure 2) is a novel semi-synthetic heterocyclic compound available in powdered form as a white crystal with a melting point of 195-197 ^°^C. R_f_ value of 0.5 was determined on TLC using 30% ethanol: Hexane as a solvent phase in hexane: ethyl acetate. With its molecular formula of C_20_H_30_N_2_O (calculated molecular weight of 314.4 g/mol). 

IR spectrum (in KBr) indicated bands at 2958 (0-H stretching), 3575 (N-H stretching), 1603 (C=C) and 1676 (C=O). 

The diterpene structure was confirmed using^ 1^H NMR (400 MHz, CDCl_3_, δ ppm) and ^13^C NMR (400 MHz, CDCl_3_, δ ppm) by the presence of ten cyclic protons at δ_H _2.05 (2H, m, H2), 1.43 (2H, m, H1), 1.27-37 (1H, m, H6), 5.22(1H, m, H3), 1.41 (2H, m, H7), 1.38 (2H, m, H10), 1.40 (1H, m, H8) and the ethylene bridge was established by δ_H _2.50 (2H, m, H12) and 1.50-1.52 (1H, m, H11), with corresponding carbon signals at δ_C _19.8 (C12) and 36.3 (C11) respectively. According to NMR spectral data obtained. four methyl signatures at δ_H _1.64 (3H, s, H18), 0.78 (3H, d, J=5.1Hz, H17), 0.86 (3H, s, H20) and 1.03 (3H, s, H19), with carbon signals at δ_C_18.3 (C18), 16.1 (C17), 19.6 (C20) and 20.6 (C19), respectively, and two methylene protons at δ_H_6.71 (1H, s, H14), and 5.22 (1H, m, H3), with carbon signal at δ_C_149.5 (C14), and 120.2 (C3) respectively, including an -OH, was also noticed at δ_H_11.20 (1H, s, H15), with corresponding carbonyl carbon signal at δ_C_161.5 (C15) and -H at 16^th^ position was also noticed at δ_H_7.65 (1H, s, H16) with corresponding carbonyl carbon signal at δ_C_139.3 (C16).

The INEPT and HMBC correlation spectrums provided more understanding of the compound structural features. HMBC correlation spectra indicated protons: Carbon resonating correlation as: δ7.65: δ19.84; δ11.67: δ149.5 and δ6.71: δ 19.84. Hence, the compound could be identified as Compound 1 (SP-38). ^1^H and ^13^C spectral data of Compound 1(SP-38) is shown in Table 1 below.

The physicochemical constants of Compound SP-38 were determined as shown in Table 2 below.

***Biological evaluation***

*Acute toxicity test*

Not any toxic symptoms or death were determined on the oral dosage of isolated SP-38 at 2000 mg/kg in rats. Thus, it discloses that the synthesized SP-38 compound was found to be relatively safe.

*Clinical assessment of arthritis and arthritis index*

All the FCA-injected groups showed significant arthritis indices compared to the normal control groups from days 4 to 28 at *P*<0.05. The arthritis indices of the standard (MTX) group and SP-38 20 mg/kg group only were markedly reduced on day 20 at *P*<0.05 compared to the arthritic control group control. Also, animals treated with standard (MTX), SP-38 20 and 10 mg/kg showed a significant decrease in arthritis index scores on day 24 compared to the arthritic model group at *P*<0.05. The results showed that after oral administration of SP-38, the higher two doses (20 and 10 mg/kg) used and the standard (MTX) could markedly improve various symptoms of arthritic rats on day 28 at *P*<0.05 with insignificant comparable differences. **I**n comparison with the standard control group, the arthritis scores of the SP-38 lower dose (5 mg/kg) treated groups were not significantly reduced on days 20, 24, and 28 at *P*<0.05. Notably, all SP doses used (SP-38 10 mg/kg, SP-38 10 mg/kg SP-38 5 mg/kg doses showed insignificant results compared to the standard drug (MTX)(Figure 3).

*Rat paw volume*

The volumes of the right hind paw were significantly increased in all groups of rats injected with FCA compared to the healthy normal control group from day 4 to day 28 with *P*<0.05. The percentage change in paw volume of all arthritic-induced groups differed significantly from the normal control group on days 12 to 28 of the experiment at *P*<0.05. The standard (MTX) only significantly reduced the paw volume; its percentage change in paw volume is significantly different from day 20 to the end of the experiment at *P*<0.05 compared to the arthritic control group and the SP-38 5 mg/kg group. After treatments with MTX and SP-38 at doses of 20 and 10 mg/kg, both paw swelling and the percentage change in paw volume were significantly different on day 24 with *P*<0.05 compared to the arthritic control group. At day 28, the SP 20 mg/kg, 10 mg/kg, and 5 mg/kg treated groups were also significant compared to the arthritic control group. Only the lower dose group (SP-38 5 mg/kg) showed significant differences in both paw volumes (less reduced) and percent change (small percentage) in paw volume compared to the standard group (MTX) from day 20 to day 28 of the experiment, while no noticeable differences were found for SP-38. 20 mg/kg and SP-38 10 mg/kg compared to standard (MTX)(Figure 4). 

*Rat ankle diameter (thickness)*

All groups of rats injected with FCA experienced significant ankle enlargement from day 4 to day 28 and significant percentage changes in ankle diameter from day 12 to day 28 compared to the normal control group. The arthritic control group showed a significant change in both ankle diameter and percentage change in diameter from day 16 to day 28 compared to all treatment groups. Only rats treated with MTX (standard drug) and SP-38 20 mg/kg showed a significant decrease in joint diameter and a significant percentage change in diameter from day 16 to 20 compared to arthritic control rats *P*<0.05. Treatment with lower SP-38 doses of 10 mg/kg and 5 mg/kg showed a significant decrease in joint diameter and a significant percent change in diameter only from day 24 to 28 compared to arthritic control rats *P*<0.05. SP-38 doses of 10 mg/kg and 5 mg/kg resulted in a significantly higher joint diameter and a significant percentage change in diameter only on days 16 and 24, while this also only occurred at SP 5 mg/kg compared to MTX (standard drug) control rats *P*<0.05. MTX (standard drug) and all doses used showed a significant reduction in ankle diameter and significant percentage changes in ankle diameter on days 24 and 28 compared to arthritic control. The lower dose group (SP-38 5 mg/kg) showed significant differences in both ankle diameter size (less reduced) and percent change (small percentage) in ankle diameter compared to the standard (MTX) group from day 20 to day 28 (Figure 5).

*Body weight*

Body weight gain in the arthritic control group was significantly lower than that of the normal control group and all treatment groups from days 16 to 28 (*P<*0.05). The body weight gain of the standard (MTX), SP-38 20 mg/kg, and SP-38 10 mg/kg treated groups were significantly higher than the arthritic control group on day 24 (*P<*0.05). However, compared to the normal control rats, animals treated with the SP-38 20 mg/kg, SP-38 10 mg/kg and 10 mg/kg showed significant weight changes on the 24^th^ and 28^th^ day. On day 28, all treatment groups showed a significant difference in body weight compared to the arthritic control group. Only the SP dose of 38 5 mg/kg showed significantly low weight on day 28 compared to the standard group (MTX)(*P***<**0.05). (Figure 6).

*Spleen index*

The spleen weight index increased significantly in all groups of rats with arthritis compared to the normal control group. The spleen weight index was observed to be restored in the standard (MTX) group and all SP-38 treated arthritic rats compared to the arthritic control. No noticeable differences were observed between the standard control group and the SP-38 treatment groups (Table 3). 

*Inflammatory biomarkers*

The arthritis control group showed a significant increase in serum levels of RF, CRP, and ESR compared to the normal control group, and the treatment groups showed a significant decrease in serum levels of RF, CRP, and ESR compared to the arthritis control group. All SP-38 treatment groups had significantly higher CRP levels compared to the standard control group. RF was significantly increased compared to the standard control group only at two doses, a 20 mg/kg dose and a 10 mg/kg dose (Table 4). 

*Liver marker enzymes*

The arthritis control group showed a significant increase in serum levels of SGOT, SGPT, and ALP compared to the normal control group. The treatment groups showed a significant decrease in serum levels of SGOT, SGPT, and ALP compared to the arthritis control group. All SP-38 treatment groups had significantly higher SGOT, SGPT, and ALP compared to the standard control group (Table 5).

*Determining the effect on hematological parameters*

The arthritic control rats showed a significant increase in white WBCs and PTLs but a decrease in RBCs, HGB, and HCT compared to Normal control. The WBCs and PTLs were decreased by treatment with standard drug (MTX) while RBCs, HGB, and HCT, were increased compared to arthritic control rats. There was a marked decrease of WBCs and PTLs (*P*<0.05) in SP-38 treated animals with 20 mg/kg dose compared to arthritic control rats. 10 mg/kg (*P*<0.05) and 5 mg/kg (*P*<0.05) only significantly decreased the WBCs and non-significant decreases in PLTs. However, RBCs and HGB were not significantly decreased in the lower doses of SP-38 10 and 5 mg/kg but the significance is observed in HCT 5 mg/kg (*P*<0.05) and 5 mg/kg compared to arthritic control rats (Table 6).

*Effect on proand inflammatory cytokines*

TNF-* α* and IL-6 pro-inflammatory cytokine expressions were significantly increased when arthritis developed in all arthritic-induced rat groups compared to Normal control (*P*<0.05). In the arthritic control group, the expression of cytokines, TNF-*α* and IL-6, were markedly elevated on day 28 when the test was performed in comparison with all other treatment groups. Post-treatment with MXT and SP-38 (20, 10, and 5 mg/kg) markedly decreases expression of both TNF-α and IL-6 at different levels compared to arthritic control with insignificance observation of their differences with the standard group (Figure 7).

*Radiological examination*

Radiological examination of the ankle revealed the following: The joint space was intact and no soft tissue swelling was observed in normal control rats. The paw tissue of the arthritic control group showed marked soft tissue swelling and bone erosion, leading to the destruction of the bony architecture (32). The MTX-treated group showed reduced soft tissue swelling and no narrowing of the joint space. In rats treated with SP-38 20 mg/kg and SP-38 10 mg/kg, joint space was reduced and soft tissue swelling was reduced. However, there was no difference in joint space between these two test dose groups and the MXT group (29)(Figure 8).

*Histopathological examination and evaluation*

Articular cartilage tissue was harvested from the right knee joint at the end of the experiment and H&E staining was performed. The normal control group indicated undamaged epidermis and dermis, lack of inflammation, cartilage erosion, and the normal architecture of the joints was intact. The arthritic control group showed severe inflammation, cellular infiltration, pannus formation, cartilage, and bone erosion leading to disruption of the articular surface and erosion of the articular cartilage compared to Normal and treated groups. The standard drug (MXT) treated group demonstrated mild inflammation. SP 20 mg/kg rats showed inflammation, moderate synovial hyperplasia, reduced cartilage and bone erosion. SP 10 mg/kg rats showed inflammation, synovial hyperplasia, cartilage and bone erosion. SP low dose 5 mg/kg rats showed severe inflammation, synovial hyperplasia, cartilage, and bone erosion as previously described (23)(Figure 9).

## Discussion

MTX is currently known as the gold standard for the treatment of RA as DMARD still poses some resistance and toxicity issues in patients, which makes scientists switch to natural products (compounds and formulations) which may be a way to safer drugs for RA remission still retain their nature with fewer side effects (24, 33). In addition, the modification/synthesis of natural compounds is thought to have paved the way to the solution and discovery of drugs with the desired effect but with various undesirable side effects (8, 9).

It has been established that rats, as an animal model of RA, have morphological characteristics similar to human diseases and can predict the efficacy of anti-arthritis drugs. Induction of arthritis using FCA yields an arthritic-induced rat model with similar disease features and histological features to human RA, characterized by rapid onset and progression of polyarticular inflammation within 10 to 14 days and in which symptoms of arthritis are observed (34, 35). Therefore, the present research attempted to evaluate the anti-arthritic activity of SP-38 synthesized from clerodane-diterpene (lactone) isolated from *Polianthia longifolia* in the FCA arthritis-induced rat model. The team hypothesized that molecular modification of lactone would result in a new anti-arthritic compound. 

When interpreting the biophysical parameters, paw swelling, body weight, and joint diameter in all FCA-treated groups were insignificantly varied from the normal control group at day 0 to the 8^th^ day. From the 12^th^ to the 28^th^ day, all FCA injected groups were remarkably varied *P*<0.05-0.001 in contrast to the normal control group similarly as observed by Javed *et al*. (36). The study found that MTX and SP-38 effectively reduced ankle diameter, arthritis score, and paw inflammation in arthritic rats from the 16^th^ to the 28^th^ day *P*<0.05-0.001.

An anti-arthritic agent was expected to control inflammation in FCA arthritis by lowering the arthritis index, which reflects the seriousness of inflammation (37). The highest arthritis index value at day 28 was recorded for the arthritic control group (8.19±0.34) which differs significantly from the treatment groups. However, the arthritis score in the MTX group and SP-38 treated groups reduced significantly from day 20 (*P*<0.05) to the 28^th^ day (*P*<0.001) in contrast to the arthritis control group (Figure 3). This decline persisted in all SP-38 groups (20, 10, and 5 mg/kg) by day 28 in a dose-dependent manner (*P*<0.05) (Figure 3).

The results showed that the paw volume of the rats in all groups except the normal control group changed significantly from day 8 to 28, similar to the trend explained by Manan *et al*. (38). The MXT control group showed a significant percentage volume change from day 20 (*P*<0.01), while the SP-38 20 mg/kg and 10 mg/kg groups showed significant differences from day 24 compared to the arthritic control group with *P*<0.05. As observed in the arthritic score for SP-38, 20 mg/kg and 10 mg/kg groups could not show significant differences in percentage change in paw volume compared to standard drug control (Figure 4).

There was a significant difference in percent change in ankle diameter in the MXT group and SP-38 20 mg/kg group at *P*<0.05 on day 16 compared to the arthritic control group and the same significant differences lasted up to the 20^th^ day. A marked decrease in ankle diameter was observed in groups treated with the standard drug (MTX) from day 16 to the end of the study compared to arthritic control rats, the same trend as reported by Gautam *et al*. (39)(*P*<0.5). The standard control and all SP-38 doses showed marked low ankle size changes in comparison with arthritis control on days 16 and 20 earlier than the SP-38 lower doses (10 mg/Kg and 5 mg/Kg) on the 24^th^ day to the 28^th^ day. Significant variation of the ankle joint diameters of arthritis control was markedly increased compared to Standard control and SP-38 20 mg/Kg rats on days 16 and 20 at *P*<0.05 similar to their percentage changes in diameter while the lower doses were less significantly elevated compared to the Standard control group. Both the percentage and the change in ankle diameter were markedly elevated in the SP-38 10 mg/Kg dose groups only on day 16 in contrast to the Standard control group control while the SP-38 10 mg/Kg dose group large size diameter increased from day 16 to day 28 (Figure 5).

The gain in body weight of the arthritic groups was markedly significantly lower than that of the group normal control group from day 16 up to the last day of the experiment (*P*<0.05). The body weight of the SP-38 treated groups increased significantly from day 24 to 28 compared to the arthritic control groups (Figure 6). The less weight gain in rheumatism is explained to be caused by rheumatoid cachexia which is characterized by loss of appetite due to increased production of cytokines. This small weight loss indicates less effect of the dosed drug on body weight loss or gain in comparison with both the Standard control and Arthritic control groups (40, 41)(Figure 6).

Since the spleen is one of the important organs of the immune system, its index was increased in the arthritic control group compared to the normal control group. In the MTX and SP-38 treated groups, the spleen index was observed to be between the normal control group and the arthritic control group, indicating the immunosuppressive effect of the drug and thus showing a regulatory effect on the immune system (42, 43). The standard control (MTX) group and all SP-38 doses spleen index were significantly higher compared to the Normal Control group due to the effect of inflammatory agent (FCA) and were significantly low compared to the arthritic Control group due to the immunosuppressive effect of the drugs at *P*<0.01 (Table 3).

Anemia is one of the clinical features of RA and when it is significantly restored indicates the remission of the disease (44). The study found that the level of RBCs, HGB, and HCT levels were significantly reduced in the arthritis control group while WBCs and PLT counts were increased compared to the normal, while MXT treatment group and all SP-38 dosed groups had significantly decreased WBCs and PLTs and increased HCT and RBCs along with HGB in contrast to arthritis control, as shown in many studies using the adjuvant-induced arthritis model (Table 6)(25, 45, 46). The standard control (MTX) and the SP-38 higher dose were found to have remarkable enhancing hematological parameters and lower than other lower doses. 

Serum CRP and RF are the systemic inflammatory biomarkers that indicate active state RA hence their reduction indicates the decrease of RA disease activeness (44). At the same time, the ESR is known to be a non-specific indicator of inflammation and is described to be used in inflammation estimation in different recent RA research (26), Normal control group indicated the significantly smallest level of both CRP and RF (4.17±0.75 and 9.03±1.17, respectively) compared to all other FCA-induced groups, which signifies the inflammation induction to the animals. The results showed that the marked highest CRP level (25.98±4.00 mg/dl) occurred in the arthritic control group, while rats treated with MTX and SP-38 (20, 10, and 5 doses) showed reduced CRP levels, 8.01±1.67 and (16.00±2.83, 18.17±2.14 and 19.17±2.48), respectively compared to the Normal control group (4.17±0.75) similarly to some reported results (47). Higher RF values (28.97±5.71 IU/ml) were observed in rats with FCA-induced arthritis compared to all other groups due to activation of the immune system throughout the pathological condition as a response to disease or sustained stimuli as inflammation develops (48). While the Standard and SP-38 treatment groups, except the lower dose (5 mg/kg), showed relatively lower RF levels, indicating a marked remission of inflammation. A similar trend was observed in the investigation of ESR from the Normal control group and the treatment groups. The MXT-treated group had significantly reduced ESR levels and all doses of SP-38 used the ESR levels were in a dose-dependent manner (*P*<0.05) (Table 4). 

This study demonstrated a significant decrease in TNF- and IL-6 expression at SP-38 higher doses in contrast to an arthritic control group of animals as reported in previous studies (14, 49). In FCA-induced arthritis, SP-38 mediates its anti-arthritic effects by down-regulating pro-inflammatory cytokines. SP-38 significantly reduced TNF and IL-6 levels when administered orally at doses of 20 and 10 mg/kg thus supporting its anti-arthritic potential (Figure 7).

The observed changes were well suppressed by two higher doses of MTX and SP-38 as shown in Figure 8. The disappearance of tissue enlargement due to the inflammation observed in the ankle area resulted in no difference in the MTX group and the SP-38 treated groups SP-38 20 mg/kg as similarly observed in a safranal study (27). It is assumed that the inflammatory process is still in its final stages and resolution is not yet complete (Figure 8).

Consistent with the biochemical parameters and radiological analysis, histological examination showed that SP-38 treatments showed a profound impact on maintaining structural integrity in comparison with arthritic control rats. The results of our study are comparable to the results of previous studies (27). The results showed that FCA causes significant inflammatory cell infiltration, pannus formation, and bone erosion. The standard treatment showed good progress in the evaluated histopathological parameters. The outcome showed a noticeable reduction in inflammatory cell infiltration in the paw of rats as well as a reduction in edema at higher doses of SP-38 (49). However, the intervention with MTX and SP-38 showed an advancement in the disease status as indicated in histological observations. 

The remarks of our study are similar to the findings of previous investigations done by *Cellat et al.* (27). The results demonstrated that FCA causes significant infiltration of inflammatory cells, pannus formation, and bone erosion. MTX treatment showed an improvement in the histopathology parameters evaluated. The outcomes indicated a remarkable reduction in inflammatory cell infiltration in the rat paws along with reduced edema of SP-38 higher doses as demonstrated by a study on hexane-insoluble fraction from Plantago (50). However, intervention with MTX and SP-38 highest dose showed improvement in the disease condition as evidenced by histologic observations. While a light microscope verified the accumulation of a great number of inflammatory cells, a fragment of eroded cartilage and pannus formation was distinguished in arthritic control and the lower doses. A tissue sample from ankle joints in the Normal group and the standard drug (MXT) showed the absence of pannus formation, although cartilage with infiltration of inflammatory cells was observed. In addition, the radiograph and microscopical examination of the joints from the Normal group showed normal cartilage without inflammatory cell infiltration. The standard drug group did not show any high-level pathological changes in the joint, having only slight infiltration of inflammatory cells (Figure 8).

## Conclusion

Based on our results, it is evident from the present study that SP-38 has noticeable anti-arthritic potentials: it significantly reduced arthritis severity as verified by decreased arthritis score, spleen indexes, ankle swelling, paw volume, inflammatory biomarkers, and possible increased body weight gain. It played an anti-inflammatory role by inhibiting TNF- and IL-6 while reducing inflammatory cell infiltration, synovial hyperplasia, and bone and cartilage destruction. Collectively, the findings obtained from this study reveal the anti-arthritic activity of SP-38 against FCA-induced arthritis in Wister rats. The antioxidant mechanism of SP-38 in arthritic illnesses has to be studied in future research.
